# Evaluating the prognostic performance of a polygenic risk score for breast cancer risk stratification

**DOI:** 10.1186/s12885-021-08937-8

**Published:** 2021-12-20

**Authors:** Maria Olsen, Krista Fischer, Patrick M. Bossuyt, Els Goetghebeur

**Affiliations:** 1 Department of Clinical Epidemiology, Biostatistics and Bioinformatics, Amsterdam University Medical Centers, Amsterdam Public Health Research Institute, Meibergdreef 9, 1105 AZ Amsterdam, The Netherlands; 2 Institute of Mathematics and Statistics, University of Tartu, Narva mnt 18, 51009 Tartu, Estonia; 3 Estonian Genome Center, Institute of Genomics, University of Tartu, Tartu, Estonia; 4 Department of Applied Mathematics, Computer Science and Statistics, Ghent University, Institute for Continuing Education Center for Statistics, Campus Sterre, S9, Krijgslaan 281, 9000 Ghent, Belgium

**Keywords:** Prognostic, Breast cancer, Polygenic risk score, Precision screening, Risk stratification, Medical test evaluation, Biomarker evaluation, Performance measures

## Abstract

**Background:**

Polygenic risk scores (PRS) could potentially improve breast cancer screening recommendations. Before a PRS can be considered for implementation, it needs rigorous evaluation, using performance measures that can inform about its future clinical value.

**Objectives:**

To evaluate the prognostic performance of a regression model with a previously developed, prevalence-based PRS and age as predictors for breast cancer incidence in women from the Estonian biobank (EstBB) cohort; to compare it to the performance of a model including age only.

**Methods:**

We analyzed data on 30,312 women from the EstBB cohort. They entered the cohort between 2002 and 2011, were between 20 and 89 years, without a history of breast cancer, and with full 5-year follow-up by 2015. We examined PRS and other potential risk factors as possible predictors in Cox regression models for breast cancer incidence. With 10-fold cross-validation we estimated 3- and 5-year breast cancer incidence predicted by age alone and by PRS plus age, fitting models on 90% of the data. Calibration, discrimination, and reclassification were calculated on the left-out folds to express prognostic performance.

**Results:**

A total of 101 (3.33‰) and 185 (6.1‰) incident breast cancers were observed within 3 and 5 years, respectively. For women in a defined screening age of 50–62 years, the ratio of observed vs PRS-age modelled 3-year incidence was 0.86 for women in the 75–85% PRS-group, 1.34 for the 85–95% PRS-group, and 1.41 for the top 5% PRS-group. For 5-year incidence, this was respectively 0.94, 1.15, and 1.08. Yet the number of breast cancer events was relatively low in each PRS-subgroup. For all women, the model’s AUC was 0.720 (95% CI: 0.675–0.765) for 3-year and 0.704 (95% CI: 0.670–0.737) for 5-year follow-up, respectively, just 0.022 and 0.023 higher than for the model with age alone. Using a 1% risk prediction threshold, the 3-year NRI for the PRS-age model was 0.09, and 0.05 for 5 years.

**Conclusion:**

The model including PRS had modest incremental performance over one based on age only. A larger, independent study is needed to assess whether and how the PRS can meaningfully contribute to age, for developing more efficient screening strategies.

**Supplementary Information:**

The online version contains supplementary material available at 10.1186/s12885-021-08937-8.

## Background

Breast cancer screening programs aim to detect tumors before they develop into symptomatic, more advanced cancers, as early detection of localized breast cancer (i.e. cancers that has not yet metastasized) is known to have a better prognosis [1]. Current screening recommendations and guidelines rely on a careful weighing of benefits - primarily a reduction in breast cancer specific mortality - against adverse events and perceived harms, such as psychological distress due to false positive mammography results, the potential of overdiagnosis, and cost [2–5].

In this context, age is still the dominant risk marker for identifying the general target group for breast cancer screening programs. However, notwithstanding a significant increase in breast cancer incidence with age [6, 7], most women do not develop breast cancer. Consequently, a majority of screened women are exposed to the associated harms without reaping the benefits [8]. A systematic review reported a cumulative proportion of false-positive screenings after 10 years of biennial screening in women aged 50–69 years that varied between 8 and 12% [9]. This justifies a quest for additional risk markers to improve currently implemented breast cancer screening programs.

Breast cancer is a heterogenic disease for which several different subtypes of genetic mutations and variations have been identified. For example, mutations in the single high-penetrance tumor suppressor gene BRCA1/2 are associated with a high relative risk of developing breast cancer. The high-penetrant genes are, however, rare and although women with a BRCA1/2 mutation have a seven times higher risk of breast cancer [10], this subtype only explains up to 10% of all breast cancers [11]. It is hence infeasible to screen the general population for such rare genetic traits and risk-markers [12, 13]; biomarkers that are more prevalent and indicative of breast cancer are needed.

In contrast to rare, high-penetrant genes, the so-called single-nucleotide polymorphism (SNPs) are genetic variants that are more commonly expressed in the general population [14, 15]. Several studies have explored the potential prognostic value of SNPs for breast cancer risk stratification, as well as for other types of cancer [16–18].

Although studies have identified more than 100 low penetrance genes as prognostic for breast cancer [19], no single SNP has shown potential for screening purposes [20, 21]. Several reasons can be identified for this absence. While SNPs lead to various unfavorable changes in the genetic transcription, translation, and epigenetic processes, their direct biological mechanisms, in regards to cancer susceptibility, are largely unknow [22]. SNPs are only weakly associated with breast cancer, typically at a (GWAS) significance threshold of 5 × 10^− 8^ [14]. It is well-known that extremely high odds ratios are needed for new potential risk factors to achieve sizeable improvements in discriminatory performance, in particular when added to existing risk factors [23–25]. Combining effects of multiple SNPs into a single polygenic risk score (PRS) may yield a more promising marker of subgroups differing sufficiently in their genetic risk of developing breast cancer [14, 26].

So far, the validity of a previously developed prevalence based PRS was mostly demonstrated through relative risks [27]. Before involving PRS in screening strategies, we need to evaluate its risk (incidence) prediction in terms of calibration (relative and absolute agreement between the observed and estimated incidence), discrimination (the ability to correctly classify women who develop breast cancer), and reclassification (the ability to correctly re-classify women who later develop breast cancer into a higher risk group, given a specified risk threshold) [28].

Next in our PRS evaluation for breast cancer screening [27], we estimate 3- and 5-year breast cancer incidence from Cox models including either age alone or PRS and age as predictors, for women without known current breast cancer nor history of breast cancer in the EstBB cohort. We assessed the prognostic performance of the models in terms of calibration, discrimination, and reclassification.

## Methods

### Study cohort

We included de-identified data from 30,901 female participants from the EstBB cohort. In brief, the EstBB is managed by the Estonian Genome Center at the University of Tartu (EGCUT) and was established to collect genetic and health information, from a large sample of the Estonian population, to advance public health [29–31].

Eligible participants were 18 years or older volunteers with Estonian nationality. Approximately 10,000 participants were recruited from 2002 to 2004. Recruitment was thereafter paused until 2006, due to financial circumstances, but continued again from 2006 to 2012 from all of Estonia (15 counties), at which the EstBB cohort had included almost 52,000 participants.

Participants were initially, non-randomly, recruited through general practitioners. Recruitment was later extended to private practices and hospitals, and special recruitment offices of the EGCUT. Recruitment was completely volunteer-based, since no direct contact with the Estonian population was allowed; participants actively signed up after hearing about the cohort study in private, at their health care institution, or through promotion at special public events and in the media [29, 30].

See Method section in the Supplementary File for more detail of the cohort.

### Eligibility

**Fig. 1:**
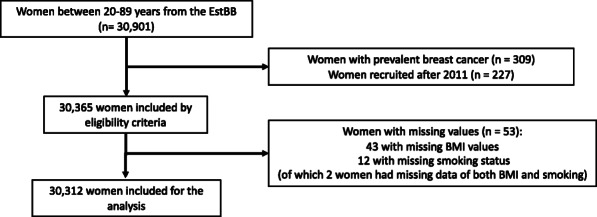
Flow diagram of participants included in the analysis

Included in this analysis were data from women from the EstBB cohort between 20 and 89 years and without a current or previous diagnosis of breast cancer at the time of cohort entry. We excluded women who had entered the cohort after 2011, as their 3- and 5-year follow-up information was not yet available at the time of analysis (Fig. 1).

Information on all variables used in this analysis (smoking status, educational level, prevalent comorbidities, age, BMI, and PRS) was initially collected on the day of recruitment through standardized interviews and questionnaires, blood samples, and from existing medical records. Diseases were classified according to the international Classification of Diseases (ICD-10) [29, 30].

The selection of SNPs and the development of PRS follow advanced and complex methods that have been described in detail elsewhere [14, 15]. In the Supplementary File we summarize the overall developmental approach of the prevalence based PRS (metaGRS_2_) used in this analysis [27].

Follow-up information on incident breast cancers and deaths was collected through biennial linkage to the Estonian Health Insurance Fund, the Estonian Causes of Death Registry, and the Estonian Cancer registry. Every recorded diagnosis of breast cancer was confirmed by an oncologist. The last linkages used in this analysis were performed in December 2015 for breast cancer and in June 2017 for death [27]. Below, we studied incident diagnosed breast cancer and death after entry into the EstBB cohort. More information about the EstBB cohort and the PRS can be found elsewhere [27, 29–32].

### Statistical analyses

Descriptive statistics for our study group are presented in Table 1. PRS results were categorized into 6 subgroups (0–25%, 25–50%, 50–75%, 75–85%, 85–95% and the top 5% PRS percentiles) as in Läll et al. [27]. A Cox proportional hazards model for incident breast cancer was then fitted with main effects of age, BMI, year of entry, 6-level categorical predictors of PRS, smoking status (never/former/current), education (less than secondary/secondary/university degree), and prevalent co-morbidities (any prevalent cancer, Type 1 diabetes, Type 2 diabetes, myocardial infarction, and coronary artery disease), together with interaction terms for BMI and age, and PRS and age. Follow-up time was measured in years from cohort entry to last linkage.

**Table 1:** Demographic and clinical characteristics of included participants

Characteristic	n (%); mean / median (SD; IQR)
Total included	30,312
Follow-up time (by June 2017)	9 / 8.59 years (2.6; 7.48–9.47)
Age	46 / 45 years (17; 32–58)
BMI	26.44 / 25.39 (5.69; 22.21–29.74)
Smokers	
Currently:	6884 (23%)
Former:	3087 (10%)
Never:	20,341 (67%)
Education	
Less than secondary:	4077 (14%)
Secondary:	17,979 (59%)
University degree:	8256 (27%)
Co-morbidities	
Mean number:	0.2336 (range: 0–5)
With 0:	24,962 (82%)
With 1:	3952 (13%)
With 2:	1117 (4%)
With 3–5:	281 (1%)

Co-morbidities are all prevalent and include (any) cancer (pcancer), type 1 diabetes (T1D), type 2 diabetes (T2D), myocardial infarction (pMI), and coronary artery disease (pCAD)

Only two covariates were statistically significant and retained in subsequent models: age and PRS. We then considered the model including only age, reflecting the current screening strategy, and another adding PRS to age as predictors. With the latter PRS-age-based model, fitted to the full data set, we estimated breast cancer specific hazard ratios with main effects for age and PRS-groups (Supplementary Table S3).

The proportionality assumption was checked using Schoenfeld’s residuals (Supplementary Fig. S1). Nested models were compared with a likelihood ratio test. We estimated cause specific hazards of ‘death without breast cancer’ and ‘breast cancer’ from Cox regressions. We call the derived model-based cumulative incidences PRSage_mInc and age_mInc, respectively.

To avoid overoptimistic incidence estimates, we evaluated each model’s performance using 10-fold cross-validation. We randomly split the data into 10 equally sized parts, of which 9 parts were used to fit the model and derived 3- and 5-year estimated incidences for the remaining 10th part test set. All 10 test-sets were then combined into the merged data set with their independently estimated incidences. This was used to evaluate calibration, discrimination, and reclassification for both PRSage_mInc and age_mInc.

Since the screening age in Estonia at the time of the analysis was 50 to 62 years [33], we constructed three age categories of women: < 50 years, 50 to 62 years, and > 62 years and combined these with the PRS groups, to evaluate calibration of the PRSage_mInc for women both in- and outside the usual screening age.

Our calibration plots show for each PRS-age subgroup the observed cumulative incidence against the mean PRSage_mInc for the same group. The observed cumulative incidence was calculated as the proportion (%) of breast cancer events within 3 or 5 years among the total number of women in each PRS-age subgroup, with corresponding 95% CI calculated using the Wilson method (Fig. 3, Supplementary Table S9 and S10). Per study design, no censoring was encountered over the periods envisaged.

Receiver operating characteristic (ROC) curves were constructed and the corresponding area under these curves (AUC) was calculated to express discrimination based on age_mInc and PRSage_mInc, for both the 3- and the 5–year time points. The 95% CI of the AUC was calculated with a non-parametric method (Delong) (Fig. 4).

Using a risk threshold of ≥1%, we classified women as high or low risk within 3 and 5 years. Separately for women with and without observed breast cancer we thus constructed reclassification tables for PRSage_mInc and age_mInc (see more detail in the Supplementary File Method Section). The net-reclassification index (NRI) followed by first subtracting the incorrect reclassifications from the current ones in each group and then adding the two proportions. The 95% CI intervals of the NRI were calculated as proposed by Pencina et al., 2008 [34] (Supplementary Table S13 and S14).

*P*-values below 0.05 were considered to indicate statistically significant differences. All statistical analyses were performed using RStudio (Version 1.1.463, 2009–2018 RStudio, Inc.)

## Results

Of the 30,901 women from the EstBB cohort between 20 and 89 years, 309 women had prevalent breast cancer at recruitment while 227 women had been recruited after 2011. Hence, a total of 30,365 women were eligible, of which 30,312 had complete data and were included in the analyses (Fig. 1). Demographic and clinical characteristics of this study group are presented in Table 1 and, per PRS-groups and age-groups, in Supplementary Table S1, S2, and Fig. S2.

The observed cumulative incidence of breast cancer in the study group was 3.33‰ (101 events) at 3 years and 6.0‰ (185 events) at 5 years (Supplementary File Table S9 and S10).

### Proportional hazards models and risk distributions

The PRS-age-based model fitted the data significantly better than the age-based model (*p*-value = 1.75e-14) with no significant improvement in model fit for the full model with all evaluated putative predictors (*p*-value = 0.25).

The estimated age-adjusted effect of the PRS on the breast cancer specific hazard followed the rank order of the PRS-groups, with HR ranging from 1.2 (95% CI: 0.81–1.84) to 4.6 (95% CI: 2.97–7.14), relative to the lowest 0–25% PRS subgroup (Supplementary Table S3).

**Fig. 2:**
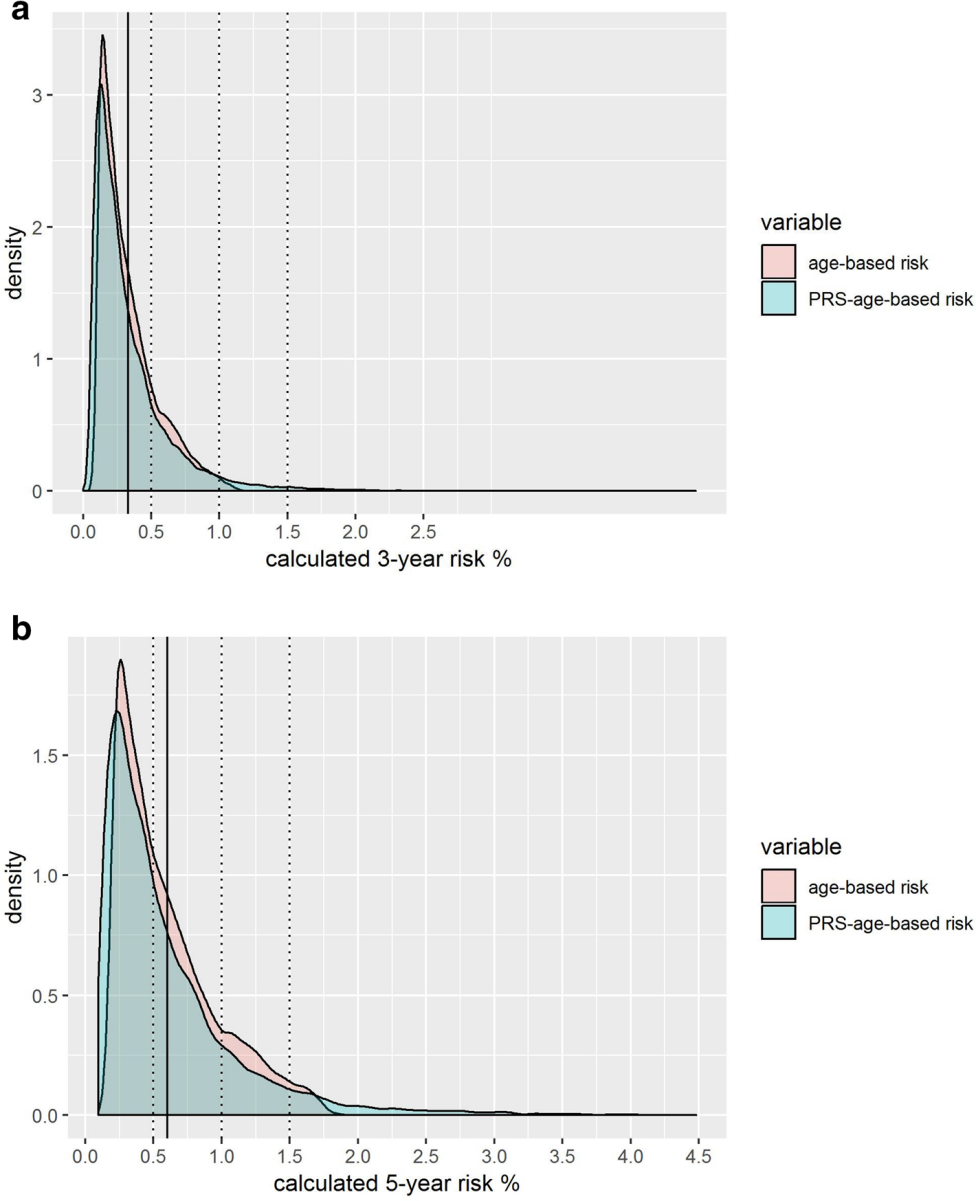
Three- and 5-years age_mInc and PRSage_mInc distributions. The figure shows the absolute incidence distributions for the age_mInc (red) and PRSage_mInc (blue). The full vertical line shows the mean PRSage_mInc and the dotted lines the 0.5, 1% and the 1.5% risk thresholds, respectively

From the Cox models, we estimated the 3- and 5-year breast cancer specific cumulative incidence for women in the cohort with the age-based model (age_mInc) and with the age plus PRS-based model (PRSage_mInc). The PRSage_mInc distribution showed a slightly wider spread than age_mInc, visible in the right-hand tail for both the 3- and 5-year estimated incidence (Fig. 2 and Supplementary Table S4 - S8).

### Prognostic performance

We evaluated the prognostic performance of PRSage_mInc in terms of calibration, discrimination, and reclassification, comparing it to age_mInc whenever appropriate.

**Fig. 3:**
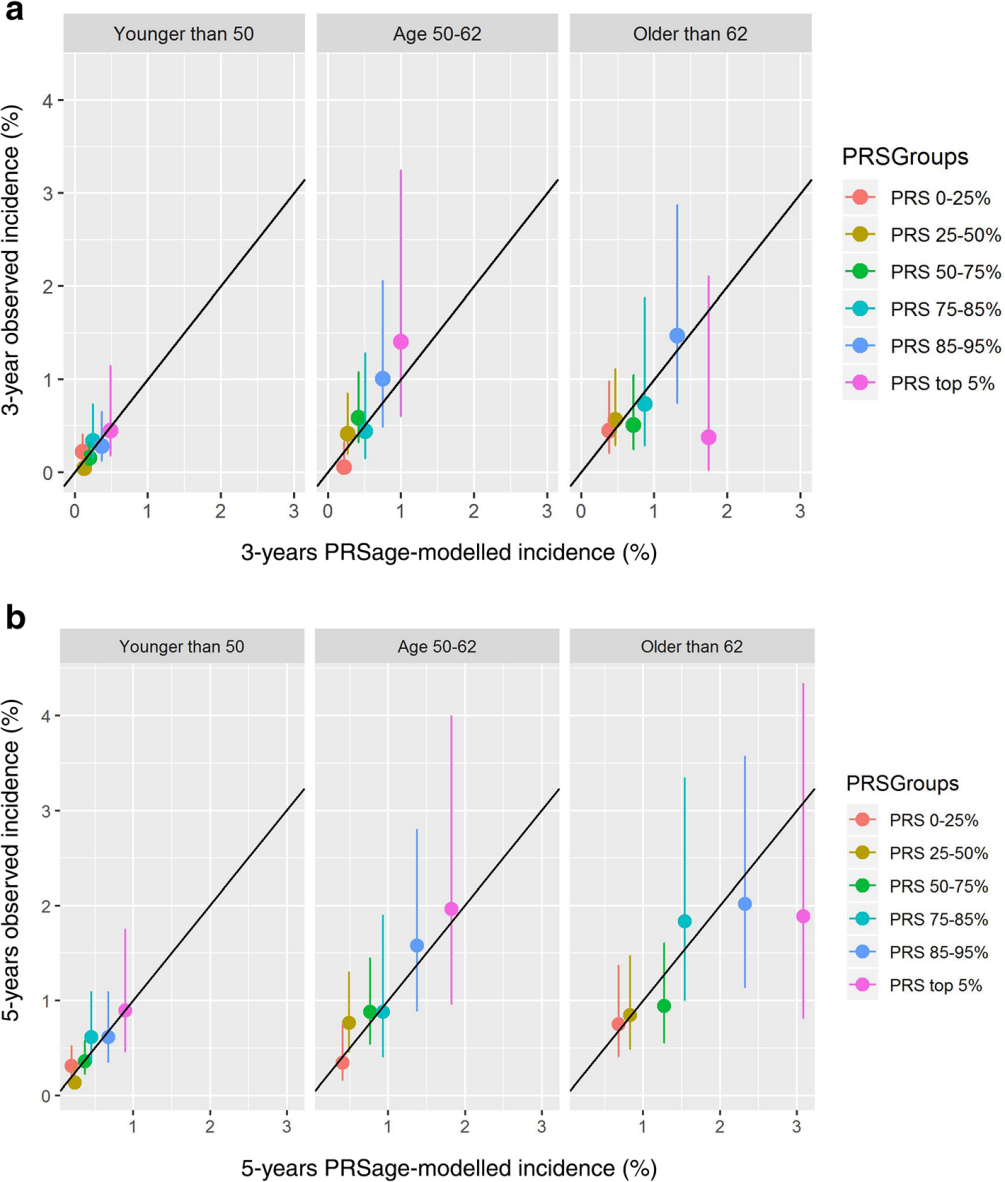
Three- and 5-year incidence calibration plots. Calibration plots with the observed cumulative incidence (incl. 95% CI) on the y-axis against the mean PRSage_mInc for each PRS-age subgroup

95% CIs for the observed incidences were wide: 1.55 to 7.35‰ and 3.39 to 11.04‰ at 3 and 5 years, respectively. Calibration varied across the PRS-age subgroups, however, the estimated incidences were still within the 95% CI for all subgroups (Fig. 3).

For women in the screening age (50–62 years), the ratio of observed cumulative incidence vs PRSage_mInc was 0.86 for women in the 75–85% PRS-group, 1.34 for the 85–95% PRS-group, and 1.41 for the top 5% PRS-group. For 5-year PRSage_mInc, these were 0.94, 1.15, and 1.08, respectively.

The approximation of PRSage_mInc to observed incidence was similar for the top 3 PRS-groups for women below 50 years and also for the age groups above 62 years, with exemption of the groups of women in the top 5% PRS-group and older than 62 years (*n* = 265), for which the PRSage_mInc was considerably overestimated. Yet, the number of breast cancer events were low in each of the PRS-age subgroups. More detail on calibration for the 3- and 5-year observed incidence and PRSage_mInc across all PRSage subgroups is shown in Supplementary Table S9 - S12.

**Fig. 4:**
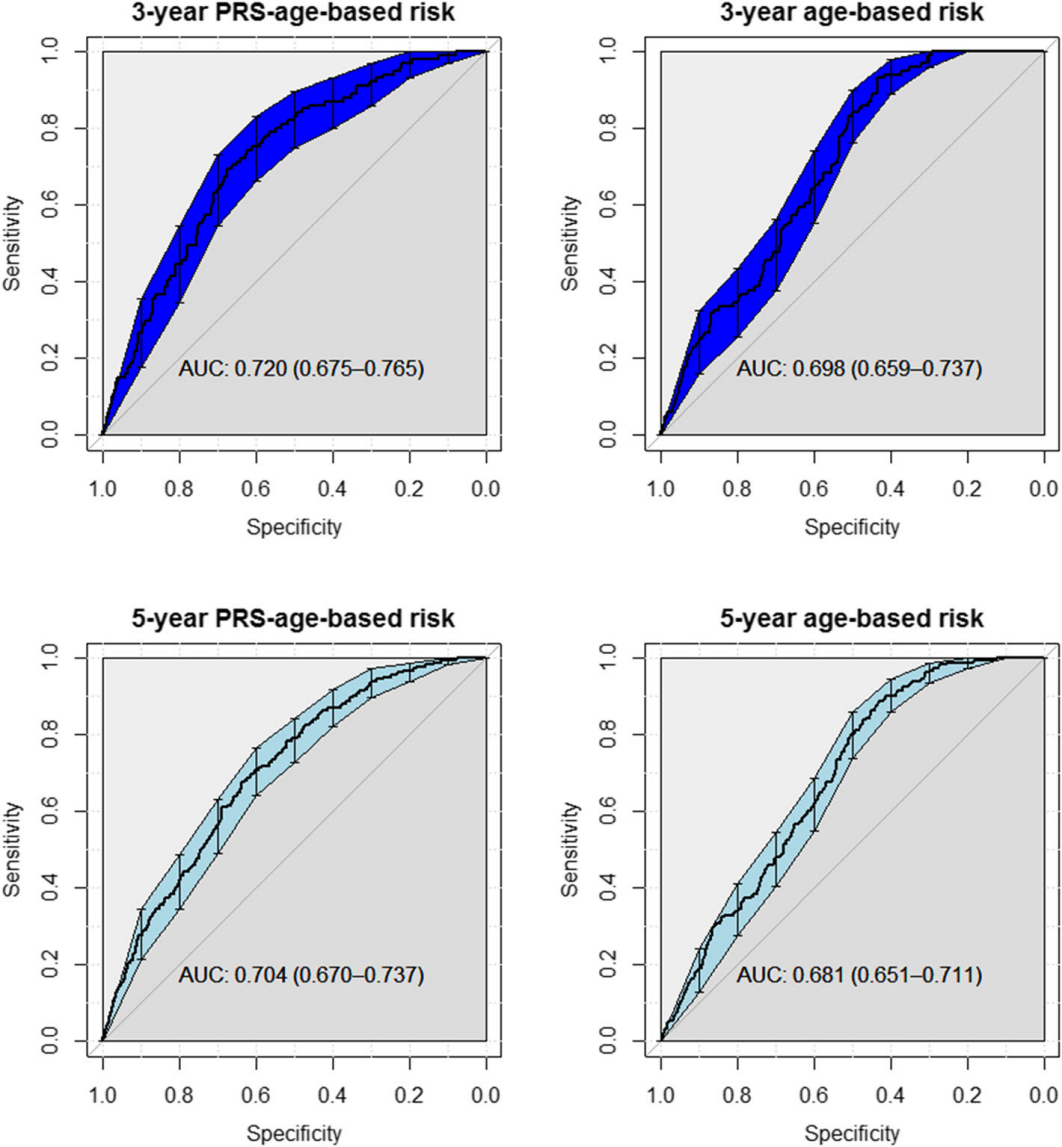
ROC and corresponding AUC of the PRSage_mInc and age_mInc

The ability of PRSage_mInc to accurately classify women with breast cancer into a high-risk category and women without breast cancer into a low-risk category was assessed by building ROC curves and estimating the corresponding AUC. The PRSage_mInc model had an AUC of 0.720 (95% CI: 0.675 to 0.765) for 3-years and 0.704 (95% CI: 0.670 to 0.737) for 5-years, respectively, just 0.022 and 0.023 higher than for the age_mInc model. Neither increment was statistically significant (*p*-values 0.23 and 0.11 respectively, calculated with the Delong test) (Fig. 4).

We constructed risk classification tables for age_mInc and PRSage_mInc, using a risk threshold of 1% for both 3- and 5-years (Supplementary Table S13 and S14), and scrutinized reclassification through the use of PRSage_mInc rather than age_mInc.

The 3-year risk reclassification showed that 13% of women with breast cancer (13/101) were correctly reclassified into a high risk-group while 1% (1/101) was incorrectly reclassified by PRSage_mInc. This resulted in a net-improvement of 12% correctly reclassified women with breast cancer. Among women without breast cancer (30,211), PRSage_mInc reclassified more women incorrectly as high risk (3%) than correctly as low risk (0.4%), resulting in a net-loss of 2.6% of incorrect upward reclassifications (Supplementary Table S13). The net reclassification index (NRI) was 0.09 (95% CI: 0.02–0.16, *p* = 0.01).

For the 5-year risk, PRSage_mInc correctly reclassified 15% of women with breast cancer as high-risk (27/185) but also incorrectly reclassified 10% (18/185) downwards, leading to a net improvement of 5% correct reclassifications of women with breast cancer. In women without breast cancer (30,127), 6.7% were incorrectly reclassified upwards, while 6.9% were correctly reclassified downwards (Supplementary Table S14), resulting in a net improvement of 0.2% and an NRI of 0.05 (95% CI: − 0.02 - 0.12, *p* = 0.17).

## Discussion

We evaluated the incremental prognostic value of adding PRS as a biomarker to age, in a Cox proportional hazards model, for screening of the general population. We found that adding PRS to age significantly improved model goodness-of-fit. The 3-year and 5-year PRSage_mInc were well calibrated. Overall improvements in terms of discrimination and reclassification were, however, modest.

The large number of participants in the EstBB cohort, together with the genotyping data that were available for each participant and the direct linkage to several Estonian population registries [29, 30, 35], are key strengths of this study. Even though events per category were relatively limited, we estimated absolute 3- and 5-year incidence from these data, in which all observation times were uncensored up to 5 years of follow-up. To our knowledge, no other study has examined absolute risk estimates with a similar design. Instead, other PRS evaluations have relied on input from external sources to convert relative to absolute risks [20, 21, 36–40].

A number of limitations to our study should also be acknowledged. The EstBB participants were non-randomly recruited, primarily through general practitioners and other medical sites. Consequently, the included women are younger, slightly more resourceful and better educated than the general Estonian population [29, 30]. The analysis was furthermore retrospective meaning that the data used for the analysis, primarily the event of breast cancer, was collected before this study was planned. This could limit generalizability of our results and potentially introduce a selection bias, respectively. As breast cancer incidence is higher in the middle to older generation, the incidence in our cohort may be underrepresented. However, the observed incidence of breast cancer in our analysis is in accordance with Estonian breast cancer statistics [41]. The cohort was, moreover, prospectively recruited with a dedicated purpose of performing health-research and no participant was lost to follow-up.

Errors in data registration, such as missing or misclassified events of breast cancer or death, could also exist. Yet, considering the large sample size and the link to several, different and well-established registries, we believe that the number of such registry-errors is unlikely to have a major impact our results.

Though no statistically significant interaction between age and the PRS was observed, we cannot rule out a stronger discriminating effect on breast cancer risk from the PRS in younger than in older women. The number of breast cancer events in the EstBB cohort may have been too small for the formal test to have sufficient power. Such a PRS-age interaction has been reported in the PRS evaluation by Mavaddat et al., 2015 and 2019 [19, 21].

Three and 5 years are relatively short time horizons for evaluating model-estimated breast cancer incidences, in particular because relatively few incident events have emerged in the cohort. This led to large uncertainty intervals for the top 5% PRS-groups primarily. Yet we believe that these time points are particularly relevant in the evaluation of the PRS for potential use in current practice. As the Estonia screening program invites women for screening every second year and the EstBB cohort had full follow-up until 5 years, cohort entry could mimic a ‘first screen’ in our sample. Indeed, no women in our sample had known current or previous breast cancer and the 3- and 5-year time points roughly correspond to the timing of two screening rounds following cohort entry.

Several studies have shown that women with a high PRS are at increased risk of breast cancer [19–21, 37–39, 42]. One of the most recent publications was made by Läll and colleagues, from the Estonian Genome Center. They developed and compared seven different types of breast cancer PRS, of which one (metaGRS_2_) showed a HR of 4.2 (95% CI: 2.8–6.2), when women from the Estonian Biobank Cohort (EstBB) in the 95th percentile (the top 5% PRS-group) were compared to women in the lowest 50th percentile PRS-group [27]. In agreement with results from Läll et al. and other PRS evaluations [19, 21, 27, 37–39, 42], the PRS was found to be moderately to strongly associated with breast cancer, primarily when comparing the top 3 percentile PRS-groups to the lowest (0–25%) PRS-group.

In terms of calibration, PRSage_mInc approximated the observed incidence relatively well for each PRS-age subgroup, as only one exception, the top 5% PRS in women older than 62 years, was considerably overestimated at both time points. This overestimation may own to the low sample size of this subgroup. Some under- and overestimation is expected at both tails of the PRSage_mInc. Calibration was more accurate for the highest 75–100% PRS-group than for the lowest 0–25% PRS-group. This too could be explained by the considerably higher observed incidence in the highest 75–100% PRS-group, with similar total sample size.

The AUC estimates the percentage of (randomly selected) pairs of women with and without breast cancer where the woman with breast cancer has the higher risk prediction, here age_mInc and PRSage-mInc. Upon adding PRS to age, the increase in AUC was limited to 2–3% more pairs assigning higher risk to the breast cancer case. Although the AUC in our analysis are slightly higher than in other PRS evaluations [21, 36–39, 42], two studies have reported similarly small improvements in AUC of 3–4% [37, 38], while two other studies reported an increase of 7–9% when adding SNPs/PRS to existing risk factors [21, 39]. Yet, the AUC does not inform on whether the small improvement could translate into sufficient discrimination of clinically defined subgroups, supporting different screening recommendations. Net-reclassification gives a more direct indication of the incremental performance upon defining potentially clinically relevant risk-groups using a threshold value. With a threshold of 1% in the reclassification we exemplified defining new PRSage-based risk groups with corresponding screening recommendations.

The 3-year PRSage_mInc showed a modest but statistically significant improvement in overall reclassification over age_mInc, whereas the improvement was smaller and non-significant for the 5-year incidence. Several other studies also showed that incremental improvements by SNPs/PRS were primarily restricted to women with breast cancer, while improvements for women without breast cancer was almost absent [20, 36, 38, 39]. Except for one smaller study, which found that women without breast cancer were also shifted downwards [37], these studies overall suggest that the benefits are primarily limited to women with breast cancer, often at a small cost for women without breast cancer. In summary, this suggests that the potential for the PRS to have sufficiently large implications for screening programs may be limited.

If and to what extent the PRS could meaningfully contribute to the development of more efficient screening strategies could not be fully answered by our data and should be further evaluated. The wave of incoming data from 150.000 new participants in the EstBB cohort [43–45] will enable a more refined and a more precise evaluation of the estimated incidences in the PRS-age-subgroups. The Estonian Genome Center is also planning a pilot project to explore if the PRS can identify subsets of women outside the screening age, who could be recommended (mammography) screening at an earlier or older age (e.g. age 45–49 and 70–75 years), since the benefit of using PRS for women within the usual screening age was modest.

Future evaluations may consider different risk thresholds, use microsimulation models to explore how risk-based screening could bring benefits for the high-risk group, in terms of breast cancer specific- and overall mortality. It may also document corresponding potential harms.

To our knowledge, no countries have implemented genetic testing for screening purposes. Yet advanced technology for genotyping is already in place in Estonia [43], and such test only requires a single blood sample collected at any given time. In this setting, the economic costs for detecting SNPs would not appear to be a limiting factor for adding PRS to age. From a broader public health perspective, clinical- and cost-effectiveness should be evaluated in the next phases of the biomarker evaluation, together with the broader impact of implementing PRS, given the resources, ethical, and safety aspects of large-scale genetic testing [46].

For a complete biomarker evaluation, a large, pragmatic, randomized trial would bring valid and convincing evidence of clinical effectiveness. Though few such studies are conducted in biomarker research, several larger trials were recently initiated, to evaluate the feasibility and/or effect of alternative risk-based strategies, that utilizes women’s personal risk to guide at what age to start, when to stop, and how often to screen. The women’s personal risk is then calculated using PRS, among other risk factors.

One is the WISDOM study in US (ClinicalTrials.gov identifier NCT02620852). This is a prospective, randomized trial, aiming to compare the proportion of breast cancers detected (stage IIB or higher) between a control group, which receives standard of care (annual routine) screening and an intervention, i.e. risk-based, group of women between 40 to 74 years, for a time horizon of 5 years. Personal risk is calculated using 9 high penetrant genes and nearly 200 SNPs, in addition to other known risk factors including family and medical history, and breast density [47]. A second randomized study is the My Personalized Breast cancer Screening (MyPeBS), a large European international initiative (ClinicalTrials.gov Identifier NCT03672331) [48]. This trial compares the incidence rate of stage II (and higher) breast cancer in 4 years between a control group (women receiving standard of care screening according to their given national guidelines) and an intervention group of women from 40 to 70 years, who also receive alternative, personalized risk-guided screening. The risk in this study is calculated using age, family history, medical history, breast density, hormone and reproductive history, in addition to a PRS as risk factors. A non-random feasibility trial of the PRS that we evaluated has been initiated in Estonia (ClinicalTrials.gov identifier NCT03989258). As a secondary objective, this study compares the number of screen-detected breast cancer in 3 years between two cohorts of women; one that receive standard of care and another that also receives screening recommendation, according to their genetic personal risk.

## Conclusion

Our analysis of women from the EstBB showed a low observed cumulative incidence, which varied across PRS percentile groups. Adding the PRS significantly improved the fit of a Cox proportional hazards model based on age only. The derived 3- and 5-year cumulative incidence based on PRS and age approximated the observed incidence relatively well. Through cross validation, the calculated PRS-age based incidence showed modest incremental prognostic performance, compared to the age-based incidence, in terms of discriminatory ability and reclassification. The low number of breast cancer events in the study group was associated with large uncertainty intervals and further research is needed to assess whether and how PRS can contribute to more effective breast cancer screening strategies.

## Supplementary Information

**Additional file 1: Fig. S1.** Schoenfeld’s residuals for the continuous predictor age (top) and the categorized predictor PRS (bottom). **Table S1.** Demographic and clinical characteristics of PRS groups. **Table S2.** Demographic and clinical characteristics of age groups. **Fig. S2.** Age density distribution for PRS groups. **Table S3.** Breast Cancer specific hazard ratios with main effects for age and PRS-groups. **Table S4**. Descriptive statistics for 3- and 5-year PRSage-mInc and age-mInc distributions. **Table S5.** Three-year PRSage-mInc: number (proportions) above 0.5, 1, and 1.5% thresholds. **Table S6.** Three-year age_mInc: number (proportions) above 0.5, 1, and 1.5% thresholds. **Table S7.** Five-year PRSage-mInc: number (proportions) above 0.5, 1, and 1.5% thresholds. **Table S8**. Five-year age_mInc: number (proportions) above 0.5, 1, and 1.5% thresholds. **Table S9**. Three-year observed cumulative incidence and PRSage_mInc per 1000 women. **Table S10**. Five-year observed cumulative incidence and PRSage_mInc per 1000 women. **Table S11.** Three-year observed cumulative incidence and PRSage_mInc per 1000 women for the lowest 0–25%, highest 75–100%, and top 5% PRS groups. **Table S12.** Five-year observed cumulative incidence and PRSage_mInc per 1000 women for the lowest 0–25%, highest 75–100%, and top 5% PRS groups. **Table S13.** Reclassification tables of women with 1% 3-year risk threshold. **Table S14**. Reclassifications in women with 1% 5-year risk threshold.

## Data Availability

We do not have ethical approval to share individual data for Estonian Biobank. Access to the data set used and analysed during the current study can be requested via data application form, via https://www.geenivaramu.ee/en/access-biobank.

## Abbreviations

**age_mInc**: Age-modelled incidence (age_mInc). Absolute cross-validated model-based breast cancer incidences, estimated with a Cox Proportional regression model including only age as covariate.
**AUC**: Area under the curve
**CI**: Confidence intervals
**EGCUT**: Estonian Genome Center at the University of Tartu
**EstBB**: Estonian Biobank
**HR**: Hazard ratio
**IQR**: Interquartile range
**NRI**: Net reclassification index
**PRSage_mInc**: PRS-age-modelled incidence (PRSage_mInc). Absolute cross-validated model-based breast cancer incidences, estimated with a Cox Proportional regression model including PRS plus age as covariates.
**ROC**: Receiver operating characteristic
**PRS**: Polygenic risk score(s)
**SD**: Standard deviation
