# Supplementary material for: Evaluating the prognostic performance of a polygenic risk score for breast cancer risk stratification
Source: BMC Cancer. 2021 Dec 20;21:1351. doi: 10.1186/s12885-021-08937-8 (PMC8691010; doi:10.1186/s12885-021-08937-8)
Supplement: Supplementary file 1 — Additional file 1: Fig. S1. Schoenfeld’s residuals for the continuous predictor age (top) and the categorized predictor PRS (bottom). Table S1. Demographic and clinical characteristics of PRS groups. Table S2. Demographic and clinical characteristics of age groups. Fig. S2. Age density distribution for PRS groups. Table S3. Breast Cancer specific hazard ratios with main effects for age and PRS-groups. Table S4. Descriptive statistics for 3- and 5-year PRSage-mInc and age-mInc distributions. Table S5. Three-year PRSage-mInc: number (proportions) above 0.5, 1, and 1.5% thresholds. Table S6. Three-year age_mInc: number (proportions) above 0.5, 1, and 1.5% thresholds. Table S7. Five-year PRSage-mInc: number (proportions) above 0.5, 1, and 1.5% thresholds. Table S8. Five-year age_mInc: number (proportions) above 0.5, 1, and 1.5% thresholds. Table S9. Three-year observed cumulative incidence and PRSage_mInc per 1000 women. Table S10. Five-year observed cumulative incidence and PRSage_mInc per 1000 women. Table S11. Three-year observed cumulative incidence and PRSage_mInc per 1000 women for the lowest 0–25%, highest 75–100%, and top 5% PRS groups. Table S12. Five-year observed cumulative incidence and PRSage_mInc per 1000 women for the lowest 0–25%, highest 75–100%, and top 5% PRS groups. Table S13. Reclassification tables of women with 1% 3-year risk threshold. Table S14. Reclassifications in women with 1% 5-year risk threshold. [file 12885_2021_8937_MOESM1_ESM.docx]

# Supplementary File

## Methods

**Description of participants in the EstBB**

By 2012, the number of participants in the EstBB cohort corresponded to approx. 5% of the adult Estonian population; 66% were women. The majority is of Estonian ethnicity (81.2%) but the cohort also includes Russian (15.4%), Ukrainian (1.3%), and Belarusian (0.6%) ethnicities. According to the Estonian census report from 2000(1), the EstBB cohort overall represents the general population well although there is an overrepresentation of women (by approx. 10%), of younger- and middle-age generations, and of people with a higher and professional secondary educational level(2,3).

**PRS development**

Läll and colleagues first considered four different PRS (GRS) for the EstBB cohort. These were developed based on the principle that the individual effects of identified breast cancer SNPs, each weighted by their corresponding logistic beta-coefficients (most often from GWAS), can be linearly combined into a single PRS-value. A person’s individual PRS-value is then the weighted sum of the SNPs that this person carries(4,5).

The first two PRS, named GRS_70_ and GRS_75_, were developed for the EstBB cohort using two previously identified and published sets of SNPs(6,7): one of 70 SNPs and another set of 75 SNPs, respectively. For the other two PRS, one named GRS_ONCO_ and another name GRS_UK_, Läll and colleagues first identified and selected two sets of SNPs, using GWAS summary statistics published from the Breast Cancer Association Consortium and the UK Biobank, respectively.

As a last step, they combined the four PRS (GRS_70_, GRS_75_, GRS_ONCO_, and GRS_UK_) to derive another three PRS, referred to as meta-PRS. In brief, these were based on the weighted average of the four individual PRS (named metaGRS_4_), the weighted average of the three strongest associated PRS (metaGRS_3_), and the weighted average of the two strongest associated PRS (named metaGRS_2_). Out of the total seven PRS, metaGRS_2_ showed the strongest association with breast cancer and was selected for this analysis. More detail about the selection of SNPs and the development of PRS can be found in the full report and Supplementary Files of Läll et. al(5).

**Figure S1.** Schoenfeld’s residuals for the continuous predictor age (top) and the categorized predictor PRS (bottom)


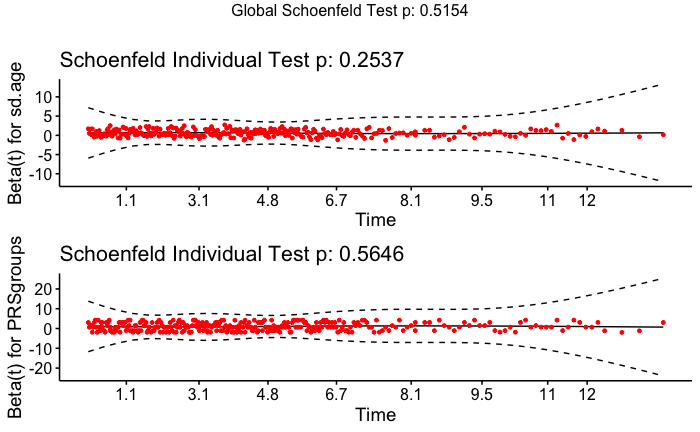


**Reclassification**

Hence, within the group of women with breast cancer the absolute number and proportions of correctly reclassified women (i.e. women moving from an age_mInc below 1% to a PRSage_mInc ≥1%) and incorrectly reclassified women (i.e. women moving from an age_mInc ≥1% to an PRSage_mInc below 1%) were calculated. This was likewise calculated for the group of women without breast cancer, but with the opposite direction for correct reclassifications (i.e. women moving from an age_mInc ≥1% to a PRSage-mInc below 1%) and incorrect reclassifications (i.e. women moving from an age_mInc below 1% to a PRSage_mInc ≥1%).

## Results

**Table S1.** Demographic and clinical characteristics of PRS groups

| PRS group | 0-25% | 25-50% | 50-75% | 75-85% | 85-95% | Top 5% |
| --- | --- | --- | --- | --- | --- | --- |
| **Number of women** | 7584 | 7584 | 7575 | 3022 | 3031 | 1516 |
| **age (mean)** | 45.74 | 46.05 | 45.95 | 45.76 | 45.90 | 45.65 |
| **BMI (mean)** | 26.50 | 26.38 | 26.50 | 26.29 | 26.45 | 26.48 |
| **PRS* (mean)** | -0.76 | -0.20 | 0.19 | 0.51 | 0.78 | 1.26 |
| **Number of comorbidities (mean)** | 0.24 | 0.24 | 0.23 | 0.24 | 0.22 | 0.23 |
| **Education: less than secondary (count)** | 1001 | 1014 | 1040 | 410 | 414 | 198 |
| **Education: secondary (count)** | 4448 | 4458 | 4517 | 1828 | 1798 | 930 |
| **Education: university degree (count)** | 2135 | 2112 | 2018 | 784 | 819 | 388 |
| **Smoking currently (count)** | 1653 | 1691 | 1734 | 732 | 733 | 341 |
| **Smoking former (count)** | 750 | 824 | 771 | 297 | 283 | 162 |
| **Smoking never (count)** | 5181 | 5069 | 5070 | 1993 | 2015 | 1013 |

***Standardized**

**Table S2.** Demographic and clinical characteristics of age groups

| Age group | Younger than 50 | | 50-62 years | Older than 62 |
| --- | --- | --- | --- | --- |
| Number of women | 17937 | | 6891 | 5484 |
| age (mean) | 34.39 | | 55.59 | 71.27 |
| BMI (mean) | 24.82 | | 28.63 | 29.02 |
| PRS* (mean) | 0.00 | | 0.00 | 0.00 |
| Number of comorbidities (mean) | 0.04 | | 0.29 | 0.79 |
| Education: less than secondary (count) | | 1419 | 816 | 1842 |
| Education: secondary (count) | 11111 | | 4277 | 2591 |
| Education: university degree (count) | 5407 | | 1798 | 1051 |
| Smoking currently (count) | 5147 | | 1435 | 302 |
| Smoking former (count) | 1854 | | 814 | 419 |
| Smoking never (count) | 10936 | | 4642 | 4763 |
| Breast cancer events in 3 years | 34 | | 33 | 34 |
| Breast cancer events in 5 years | 66 | | 58 | 61 |

***Standardized**

**Figure S2.** Age density distribution in PRS subgroups


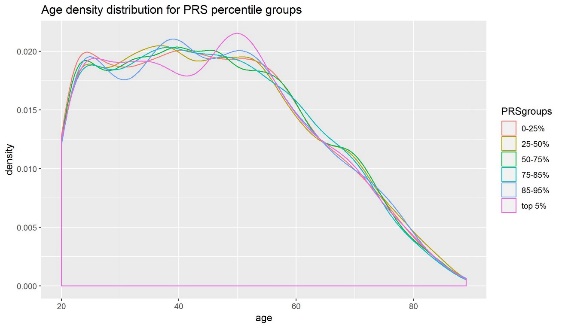


**Table S3.** Breast cancer specific hazard ratios with main effects for age and PRS-groups

| **Variable** | **Age-adjusted HR**  **exp(coef)** | **Lower**  **95% CI** | **Upper**  **95% CI** |
| --- | --- | --- | --- |
| Age (standardized)* | 1.83 | 1.63 | 2.05 |
| 0-25% | Reference | - | - |
| 25-50% | 1.22 | 0.81 | 1.84 |
| 50-75% | 1.88 | 1.30 | 2.7 |
| 75-85% | 2.32 | 1.50 | 3.58 |
| 85-95% | 3.45 | 2.31 | 5.13 |
| Top 5% | 4.60 | 2.97 | 7.14 |

* SD of age is 17

**Table S4.** Descriptive statistics for 3- and 5-year PRSage_mInc and age_mInc distributions

| Statistics | 3-year PRSage_mInc  (mean %) | 3-year age_mInc  (mean %) | 5-year PRSage_mInc  (mean %) | 5-year age_mInc  (mean %) |
| --- | --- | --- | --- | --- |
| Min. | 0.05 | 0.1 | 0.09 | 0.19 |
| 1st Qu. | 0.14 | 0.17 | 0.26 | 0.32 |
| Median | 0.24 | 0.27 | 0.44 | 0.5 |
| Mean | 0.33 | 0.33 | 0.6 | 0.6 |
| 3rd Qu. | 0.42 | 0.43 | 0.76 | 0.79 |
| Max. | 2.78 | 1.16 | 4.48 | 1.85 |

**Table S5.** Three-year PRSage-mInc: number (proportions) above 0.5%, 1%, and 1.5% thresholds

| PRS group | No. of women (n) | No. of women above 0.5% PRSage_mInc (%) | No. of women above 1% PRSage_mInc (%) | No. of women above 1.5% PRSage_mInc (%) |
| --- | --- | --- | --- | --- |
| 0-25% | 7584 | 147 (1.94) | 0 | 0 |
| 25-50% | 7584 | 462 (6.09) | 0 | 0 |
| 50-75% | 7575 | 1544 (20.38) | 85 (1.12) | 0 |
| 75-85% | 3022 | 890 (29.45) | 116 (3.84) | 0 |
| 85-95% | 3031 | 1537 (50.71) | 491 (16.2) | 135 (4.45) |
| top 5% | 1516 | 1015 (66.95) | 423 (27.9) | 187 (12.34) |
| Total | 30312 | 5595 (18.46) | 1115 (3.68) | 322 (1.06) |

**Table S6.** Three-year age_mInc: number (proportions) above 0.5%, 1%, and 1.5% thresholds

| PRS group | No. of women (n) | No. of women above 0.5% age_mInc (%) | No. of women above 1% age_mInc (%) | No. of women above 1.5% age_mInc (%) |
| --- | --- | --- | --- | --- |
| 0-25% | 7584 | 1406 (18.54) | 55 (0.73) | 0 |
| 25-50% | 7584 | 1484 (19.57) | 63 (0.83) | 0 |
| 50-75% | 7575 | 1445 (19.08) | 57 (0.75) | 0 |
| 75-85% | 3022 | 567 (18.76) | 17 (0.56) | 0 |
| 85-95% | 3031 | 573 (18.9) | 25 (0.82) | 0 |
| top 5% | 1516 | 276 (18.21) | 11 (0.73) | 0 |
| Total | 30312 | 5751 (18.97) | 228 (0.75) | 0 |

**Table S7.** Five-year PRSage-mInc: number (proportions) above 0.5%, 1%, and 1.5% thresholds

| PRS group | No. of women (n) | No. of women above 0.5% PRSage_mInc (%) | No. of women above 1% PRSage_mInc (%) | No. of women above 1.5% PRSage_mInc (%) |
| --- | --- | --- | --- | --- |
| 0-25% | 7584 | 1389 (18.31) | 9 (0.12) | 0 |
| 25-50% | 7584 | 2115 (27.89) | 245 (3.23) | 0 |
| 50-75% | 7575 | 3925 (51.82) | 1271 (16.78) | 252 (3.33) |
| 75-85% | 3022 | 1904 (63) | 778 (25.74) | 260 (8.6) |
| 85-95% | 3031 | 2524 (83.27) | 1412 (46.59) | 739 (24.38) |
| top 5% | 1516 | 1490 (98.28) | 929 (61.28) | 597 (39.38) |
| Total | 30312 | 13347 (44.03) | 4644 (15.32) | 1848 (6.1) |

**Table S8**. Five-year age_mInc: number (proportions) above 0.5%, 1%, and 1.5% thresholds

| PRS group | No. of women (n) | No. of women above 0.5% age_mInc (%) | No. of women above 1% age_mInc (%) | No. of women above 1.5% age_mInc (%) |
| --- | --- | --- | --- | --- |
| 0-25% | 7584 | 3732 (49.21) | 1130 (14.9) | 204 (2.69) |
| 25-50% | 7584 | 3770 (49.71) | 1209 (15.94) | 237 (3.12) |
| 50-75% | 7575 | 3778 (49.87) | 1198 (15.82) | 196 (2.59) |
| 75-85% | 3022 | 1498 (49.57) | 456 (15.09) | 76 (2.51) |
| 85-95% | 3031 | 1502 (49.55) | 458 (15.11) | 80 (2.64) |
| top 5% | 1516 | 771 (50.86) | 228 (15.04) | 40 (2.64) |
| Total | 30312 | 15051 (49.65) | 4679 (15.44) | 833 (2.75) |

**Table S9.** Three-year observed cumulative incidence and PRSage_mInc per 1000 women

| PRS-age-group | Number of women (n) | Number of breast cancers in 3 years (n) | | 3-year observed Incidence (95% CI)* | 3-year mean PRSage_mInc | Difference | Ratio |
| --- | --- | --- | --- | --- | --- | --- | --- |
| 0-25% PRS; 18-49 years | 4499 | | 10 | 2.22 (1.21 - 4.09) | 1.06 | 1.16 | 2.09 |
| 25-50% PRS; 18-49 years | 4466 | | 2 | 0.45 (0.12 - 1.63) | 1.30 | -0.85 | 0.35 |
| 50-75% PRS; 18-49 years | 4494 | | 7 | 1.56 (0.75 - 3.21) | 2.01 | -0.45 | 0.78 |
| 75-85% PRS; 18-49 years | 1794 | | 6 | 3.34 (1.53-7.28) | 2.46 | 0.88 | 1.36 |
| 85-95% PRS; 18-49 years | 1789 | | 5 | 2.79 (1.19 - 6.53) | 3.67 | -0.87 | 0.76 |
| Top 5% PRS; 18-49 years | 895 | | 4 | 4.47 (1.74 - 11.43) | 4.87 | -0.4 | 0.92 |
| Sub-total; 18-49 years | 17937 | | 34 | 1.90 (0.88 - 4.19) | 1.95 | -0.05 | 0.97 |
|  |  | |  |  |  |  |  |
| 0-25% PRS; 50-62 years | 1750 | | 1 | 0.57 (0.03 - 3.23) | 2.20 | -1.63 | 0.26 |
| 25-50% PRS; 50-62 years | 1701 | | 7 | 4.12 (1.99 - 8.47) | 2.67 | 1.44 | 1.54 |
| 50-75% PRS; 50-62 years | 1704 | | 10 | 5.87 (3.19 - 10.77) | 4.17 | 1.7 | 1.41 |
| 75-85% PRS; 50-62 years | 683 | | 3 | 4.39 (1.49 - 12.83) | 5.09 | -0.7 | 0.86 |
| 85-95% PRS; 50-62 years | 697 | | 7 | 10.04 (4.87 - 20.58) | 7.51 | 2.53 | 1.34 |
| Top 5% PRS; 50-62 years | 356 | | 5 | 14.04 (6.01 - 32.45) | 9.96 | 4.08 | 1.41 |
| Sub-total; 50-62 years | 6891 | | 33 | 4.79 (2.24 - 10.60) | 4.03 | 0.76 | 1.19 |
|  |  | |  |  |  |  |  |
| 0-25% PRS; 63-89 years | 1335 | | 6 | 4.49 (2.06 - 9.77) | 3.83 | 0.66 | 1.17 |
| 25-50% PRS; 63-89 years | 1417 | | 8 | 5.65 (2.86 - 11.1) | 4.70 | 0.95 | 1.2 |
| 50-75% PRS; 63-89 years | 1377 | | 7 | 5.08 (2.46 - 10.46) | 7.18 | -2.1 | 0.71 |
| 75-85% PRS; 63-89 years | 545 | | 4 | 7.34 (2.86 - 18.72) | 8.67 | -1.33 | 0.85 |
| 85-95% PRS; 63-89 years | 545 | | 8 | 14.68 (7.46 - 28.7) | 13.17 | 1.51 | 1.11 |
| Top 5% PRS; 63-89 years | 265 | | 1 | 3.77 (0.19 - 21.06) | 17.46 | -13.69 | 0.22 |
| Sub-total; 63-89 years | 5484 | | 34 | 6.20 (2.89 - 13.06) | 6.96 | -0.76 | 0.89 |
|  |  | |  |  |  |  |  |
| Total (entire cohort) | 30312 | | 101 | 3.33 (1.55 - 7.35) | 3.33 | 0.00 | 1.00 |

**Legend**: Three-year observed cumulative incidence and mean PRSage_mInc, for each PRSage subgroup, per 1000 women. The difference is observed incidence minus the mean PRSage_mInc. The ratio expresses observed incidence over mean PRSage_mInc. *Confidence intervals were calculated with the Wilson method.

**Table S10.** Five-year observed cumulative incidence and PRSage_mInc per 1000 women

| PRS-age-group | Number of women (n) | Number of breast cancers in 5 years (n) | 5-year observed incidence (95% CI) | 5-year mean PRSage_mInc | Difference | Ratio |
| --- | --- | --- | --- | --- | --- | --- |
| 0-25% PRS; 18-49 years | 4499 | 14 | 3.11 (1.85 - 5.22) | 1.96 | 1.15 | 1.59 |
| 25-50% PRS; 18-49 years | 4466 | 6 | 1.34 (0.62 - 2.93) | 2.39 | -1.05 | 0.56 |
| 50-75% PRS; 18-49 years | 4494 | 16 | 3.56 (2.19 - 5.78) | 3.7 | -0.14 | 0.96 |
| 75-85% PRS; 18-49 years | 1794 | 11 | 6.13 (3.43 - 10.95) | 4.54 | 1.59 | 1.35 |
| 85-95% PRS; 18-49 years | 1789 | 11 | 6.15 (3.44 - 10.98) | 6.75 | -0.6 | 0.91 |
| Top 5% PRS; 18-49 years | 895 | 8 | 8.94 (4.54 - 17.54) | 8.95 | -0.01 | 1 |
| Sub-total; 18-49 years | 17937 | 66 | 3.68 (2.08 - 6.55) | 3.59 | 0.09 | 1.03 |
|  |  |  |  |  |  |  |
| 0-25% PRS; 50-62 years | 1750 | 6 | 3.43 (1.57 - 7.46) | 4.04 | -0.61 | 0.85 |
| 25-50% PRS; 50-62 years | 1701 | 13 | 7.64 (4.47 - 13.03) | 4.9 | 2.74 | 1.56 |
| 50-75% PRS; 50-62 years | 1704 | 15 | 8.8 (5.34 - 14.47) | 7.64 | 1.16 | 1.15 |
| 75-85% PRS; 50-62 years | 683 | 6 | 8.78 (4.03 - 19.03) | 9.32 | -0.54 | 0.94 |
| 85-95% PRS; 50-62 years | 697 | 11 | 15.78 (8.83 - 28.04) | 13.75 | 2.03 | 1.15 |
| Top 5% PRS; 50-62 years | 356 | 7 | 19.66 (9.56 - 40.02) | 18.22 | 1.44 | 1.08 |
| Sub-total; 50-62 years | 6891 | 58 | 8.42 (4.61 - 15.48) | 7.38 | 1.04 | 1.14 |
|  |  |  |  |  |  |  |
| 0-25% PRS; 63-89 years | 1335 | 10 | 7.49 (4.07 - 13.73) | 6.79 | 0.7 | 1.1 |
| 25-50% PRS; 63-89 years | 1417 | 12 | 8.47 (4.85 - 14.74) | 8.32 | 0.15 | 1.02 |
| 50-75% PRS; 63-89 years | 1377 | 13 | 9.44 (5.53 - 16.09) | 12.75 | -3.31 | 0.74 |
| 75-85% PRS; 63-89 years | 545 | 10 | 18.35 (10 - 33.44) | 15.41 | 2.94 | 1.19 |
| 85-95% PRS; 63-89 years | 545 | 11 | 20.18 (11.31 - 35.78) | 23.25 | -3.07 | 0.87 |
| Top 5% PRS; 63-89 years | 265 | 5 | 18.87 (8.09 - 43.4) | 30.85 | -11.78 | 0.61 |
| Sub-total; 63-89 years | 5484 | 61 | 11.12 (6.14 - 20.17) | 12.34 | -1.22 | 0.90 |
|  |  |  |  |  |  |  |
| Total (entire cohort) | 30312 | 185 | 6.10 (3.39 - 11.04) | 6.03 | 0.07 | 1.01 |

**Legend**: Five-year observed cumulative incidence and mean PRSage_mInc, for each PRSage subgroup, per 1000 women. The difference is observed incidence minus the mean PRSage_mInc. The ratio expresses observed incidence over mean PRSage_mInc.

**Table S11.** Three-year observed cumulative incidence and PRSage_mInc per 1000 women for the lowest 0-25%, highest 75-100%, and top 5% PRS groups

| PRS-age-group | No. women (n) | Number of events in 3 years (n) | 3-year observed incidence (95% CI) | 3 year mean PRSage_mInc | Difference | Ratio |
| --- | --- | --- | --- | --- | --- | --- |
| 0-25% PRS group |  |  |  |  |  |  |
| 0-25% PRS; 18-49 years | 4499 | 10 | 2.22 (1.21-4.09) | 1.06 | 1.16 | 2.09 |
| 0-25% PRS; 50-62 years | 1750 | 1 | 0.57 (0.03-3.23) | 2.20 | -1.63 | 0.26 |
| 0-25% PRS; 63-89 years | 1335 | 6 | 4.49 (2.06-9.77) | 3.83 | 0.66 | 1.17 |
| Total | 7584 | 17 | 2.24 (1.09-4.89) | 1.81 | 0.43 | 1.24 |
|  |  |  |  |  |  |  |
| 75-100% PRS group |  |  |  |  |  |  |
| 75-100%;18-49 years | 4478 | 15 | 3.36 (1.44-7.81) | 3.43 | -0.08 | 0.98 |
| 75-100%;50-62 years | 1736 | 15 | 8.64 (3.77-19.97) | 7.06 | 1.58 | 1.22 |
| 75-100%;50-62 years | 1355 | 13 | 9.59 (4.19-23.19) | 12.20 | -2.60 | 0.79 |
| Total | 7569 | 43 | 5.68 (2.46-13.35) | 5.83 | -0.15 | 0.97 |
|  |  |  |  |  |  |  |
| Top 5% PRS group |  |  |  |  |  |  |
| Top 5% PRS; 18-49 years | 895 | 4 | 4.47 (1.74 - 11.43) | 4.87 | -0.4 | 0.92 |
| Top 5% PRS; 50-62 years | 356 | 5 | 14.04 (6.01 - 32.45) | 9.96 | 4.08 | 1.41 |
| Top 5% PRS; 63-89 years | 265 | 1 | 3.77 (0.19 - 21.06) | 17.46 | -13.69 | 0.22 |
| Total | 1516 | 10 | 6.60 (2.47-18.05) | 8.27 | -1.67 | 0.80 |

**Table S12.** Five-year observed cumulative incidence and PRSage_mInc per 1000 women for the lowest 0-25%, highest 75-100%, and top 5% PRS groups

| PRS-age group | No. women (n) | Number of events in 5 years (n) | 5-year observed incidence (95% CI) | 5-year mean PRSage_mInc | Difference | Ratio |
| --- | --- | --- | --- | --- | --- | --- |
| 0-25% PRS group |  |  |  |  |  |  |
| 0-25% PRS; 18-49 years | 4499 | 14 | 3.11 (1.85-5.22) | 1.96 | 1.15 | 1.59 |
| 0-25% PRS; 50-62 years | 1750 | 6 | 3.43 (1.57-7.46) | 4.04 | -0.61 | 0.85 |
| 0-25% PRS; 63-89 years | 1335 | 10 | 7.49 (4.07-13.73) | 6.79 | 0.7 | 1.1 |
| Total | 7584 | 30 | 3.96 (2.18-7.23) | 3.29 | 0.67 | 1.20 |
|  |  |  |  |  |  |  |
| 75-100% PRS group |  |  |  |  |  |  |
| 75-100%;18-49 years | 4478 | 30 | 6.70 (3.66-12.28) | 6.30 | 0.40 | 1.06 |
| 75-100%;50-62 years | 1736 | 24 | 13.82 (7.09-26.95) | 12.92 | 0.90 | 1.07 |
| 75-100%;63-89 years | 1355 | 26 | 19.19 (10.15-36.33) | 21.58 | -2.36 | 0.89 |
| Total | 7569 | 80 | 10.57 (6.61-19.95) | 10.56 | 0.01 | 1 |
|  |  |  |  |  |  |  |
| Top 5% PRS group |  |  |  |  |  |  |
| Top 5% PRS; 18-49 years | 895 | 8 | 8.94 (4.54- 17.54) | 8.95 | -0.01 | 1 |
| Top 5% PRS; 50-62 years | 356 | 7 | 19.66 (9.56 - 40.02) | 18.22 | 1.44 | 1.08 |
| Top 5% PRS; 63-89 years | 265 | 5 | 18.87 (8.09- 43.4) | 30.85 | -11.98 | 0.61 |
| Total | 1516 | 20 | 13.19 (6.34-27.34) | 14.96 | -1.76 | 0.88 |

**Table S13.** Reclassification tables of women with 1% 3-year risk threshold

| Women with breast cancer  in 3 years | PRSage_mInc  ≤ 1% | PRSage_mInc  ≥ 1% | N | Re-classified with PRSage_mInc (%) |
| --- | --- | --- | --- | --- |
| age_mInc ≤ 1% | 86 | **13** | 99 | 13/101 =0.13 (13%) |
| age_mInc ≥ 1% | **1** | 1 | 2 | 1/101 = 0.01 (1%) |
| N | 87 | 14 | 101 | 0.13-0.01 = 0.12 (12%) |

| Women without breast cancer  in 3 years | PRSage_mInc  ≤ 1% | PRSage_mInc  ≥ 1% | N | Re-classified with PRSage_mInc (%) |
| --- | --- | --- | --- | --- |
| age_mInc ≤ 1% | 28991 | **994** | 29985 | 994/30211 = 0.03 (3%) |
| age_mInc ≥ 1% | **119** | 107 | 226 | 119/30211=0.004 (0.4%) |
| N | 29110 | 1101 | 30211 | 0.03-0.004=0.026 (2.6%) |

**Table S14.** Reclassifications in women with 1% 5-year risk threshold

| Women with breast cancer  in 5 years | PRSage_mInc  ≤ 1% | PRSage_mInc  ≥ 1% | N | Re-classified with PRSage_mInc (%) |
| --- | --- | --- | --- | --- |
| age_mInc ≤ 1% | 101 | **27** | 128 | 27/185= 0.15 (15%) |
| age_mInc ≥ 1% | **18** | 39 | 57 | 18/185= 0.1 (10%) |
| N | 119 | 66 | 185 | 0.15-0.1=0.05 (5%) |

| Women without breast cancer  in 5 years | PRSage_mInc  ≤ 1% | PRSage_mInc  ≥ 1% | N | Re-classified with PRSage_mInc (%) |
| --- | --- | --- | --- | --- |
| age_mInc ≤ 1% | 23473 | **2032** | 25505 | 2032/30127=0.067 (6.7%) |
| age_mInc ≥ 1% | **2076** | 2546 | 4622 | 2076/30127=0.069 (6.9%) |
| N | 25549 | 4578 | 30127 | 0.067-0.069 = -0.002 (-0.2%) |

References

1. General information - Statistics Estonia [Internet]. Accessed Jul 9 2020. Available from: https://www.stat.ee/26262

2. Leitsalu L, Haller T, Esko T, Tammesoo M-L, Alavere H, Snieder H, et al. Cohort Profile: Estonian Biobank of the Estonian Genome Center, University of Tartu. Int J Epidemiol. 2015;44:1137–47.

3. Estonian Genome Center, 2001-2011. Estonian Genome Center, University of Tartu. 2011.

4. Torkamani A, Wineinger NE, Topol EJ. The personal and clinical utility of polygenic risk scores. Nature Reviews Genetics. 2018;19581–90.

5. Läll K, Lepamets M, Palover M, Esko T, Metspalu A, Tõnisson N, et al. Polygenic prediction of breast cancer: comparison of genetic predictors and implications for risk stratification. BMC Cancer. 2019;19:557.

6. Mavaddat N, P Pharoah PD, Michailidou K, Tyrer J, Brook MN, Bolla MK, et al. Prediction of Breast Cancer Risk Based on Profiling With Common Genetic Variants. JNCI J Natl Cancer Inst. 2015;107:36.

7. Sieh W, Rothstein JH, McGuire V, Whittemore AS. The role of genome sequencing in personalized breast cancer prevention. Cancer Epidemiol Biomarkers Prev. 2014;23:2322–7.
